# Martin’s Bandage

**Published:** 1880-07-20

**Authors:** W. W. Dawson

**Affiliations:** Cincinnati


### MARTIN'S BANDAGE.

BY W. W. DAWSON, M.D., CINCINNATI.

Martin's elastic bandage is another important and valuable contribution to surgical resources, one destined to revolutionize the treatment of inflammation and congestion of the extremities.

As a dressing it is exceedingly agreeable to the patient, giving such uniform support to the part that a sense of relief and comfort attends its application. Care is demanded in its use, but it does not require that skill which is absolutely necessary for the efficient adjustment of the common bandage. After one or two lessons patients are qualified to apply it and to regulate the amount of pressure. Undue pressure is but seldom made, so exactly does the elastic structure adapt itself to the part covered.

From disease or injury a limb is engorged with blood, and we say the inflammatory process has begun; this is the initiative; this is inflammation or congestion in its simplest form. As the case progresses other changes occur, the blood vessels become enlarged and elongated, their walls thinned, the contents begin to escape, leucocytes show a tendency to migrate, to escape into the connective tissue; then come swelling, tension, effusion, plastic and purulent—the accidents which follow engorgement. By the elastic bandage the redundant blood is driven out of a limb, and by it a re-accumulation is prevented. Heretofore we attempted this by the ordinary roller, but irregular support at one place, and strangulation at another, was the too common result.

In addition to this control of the blood, the elastic bandage gives such support to the capillaries and absorbents that effusion is soon taken up, swelling disappears, and ulcers close. The action is as striking in the chronic as in the acute—in an old ulcer as in a recent erysipelas, in a sprained ankle as in an inflamed finger, in an œdema as in a phlegmonous infiltration; in short, the products of inflammation more rapidly disappear under its action than by any measure or measures heretofore in use.

Again, it is of value where mechanical support is required, as in an exhausted bursa the surfaces may be kept in contact until adhesion occurs, or in a chronic dropsy of a joint where pressure is needed to prevent the weeping of the serous membrane.

*Acute Erysipelas of the Leg.*—I applied the bandage to a case of this kind, marked by all its peculiar symptoms, redness of the skin, great tension, effusion into cellular tissue, a well developed phlegmonous state with a tendency to diffuse suppuration. Under the application of the bandage the limb, in twenty-four hours, had assumed almost its normal hue, temperature reduced, and the swelling to a very great extent disappeared. This was the first case of acute disease in which I tried it, and the result was most gratifying; a case, the natural history of which requires from two to six weeks, was cured in less than three days.

*Aborted Whitlow.*—Gross justly remarks that abortive measures have seldom succeeded in this ugly affection, for the tendency is rapidly to suppuration. With the elastic bandage both the deep and superficial varieties may be aborted. If used early suppuration will be prevented, but if time has been lost, the suppurative action may be arrested, and the case rendered comparatively insignificant—that is comparatively painless.

For the fingers and on the limbs of children, a very light bandage must be used. Finding that of Martin too heavy, I procured what is known in dental parlance as "rubber dam," and cut it into strips suited to each case. The lightest variety is sufficiently strong for use upon the fingers. Care must be exercised so as not to *prevent* but to *control* the circulation in the part.

*Sprained Ankle.*—It has long been said that a sprained ankle is more to be dreaded than a broken leg. What occurs in a case of this kind, and how does the elastic act? In a sprain the ligaments are ruptured to a degree, small blood-vessels are broken, hemorrhage occurs in the peri-articular tissues. This escaped blood is a foreign body, and so acts; it generates, however, in the structures around and within the joint an inflammation of a subacute form, and although possessing but little conservative or reparative power, it seldom tends to a destructive, to a suppurative action. The swelling is not great, but it is firm, what might be called stubborn. When the bandage is applied the change is as marked as it is gratifying; it is as efficient in the ancient as in the recent case.

In reply to the question how it acts in these cases, it may be said that it arrests at once the inter-tissual hemorrhage, and it supports the absorbents in taking up and carrying off the effused substances. A few hours are sufficient to reduce the acute case, a few days are necessary to bring about the same result in a case of long standing.

*Compound Fracture of Thumb.*—A man presented himself at my office one morning with the end of the thumb almost severed; the cut was oblique, and divided skin, muscles and bone; a small portion of skin was left on the ulnar side. I replaced the parts, and applied the "rubber dam" bandage; no suppuration occurred, and in a few days the union was complete.

*Hydrops Articuli.*—All surgeons know how difficult it is to cure these cases without resorting to intra-articular injections, a practice always hazardous. The fluid drawn off soon re-accumulates; this is repeated until the surgeon is worn out, and resorts finally to an irritating injection. The bandage here acts promptly by its mechanical compression of the hyper-secreting serous membrane.

In a case of this kind, pure hydrops articuli, not chronic arthritis, it had had no "John the Baptist" in the shape of acute inflammation of the joint forerunning it, being chronic *de novo*, I evacuated the fluid, applied the bandage, compressed the serous sac so that it could no longer weep; change occurred necessarily, no further effusion. The case was positively relieved.

*Diffuse Suppuration.*—This form of inflammation, following injury or disease, is promptly relieved by the judicious application of the bandage, the tissues are supported, the blood-vessels are compressed, proliferation and migration cease, and the effused material is absorbed.

In this condition the elastic must be frequently changed, so that the pus may be removed and the enclosed parts cleansed.

*Indolent Ulcers.*—These may be treated successfully, promptly cured, and the patient allowed to attend to his ordinary business. All practitioners know what difficulties have been encountered in the treatment of ulcers of the leg. In all plans rest is an essential element. The grafting process and the canal of Nussbaum have been most efficient, but to succeed with them the patient has to be confined to the recumbent posture. What a comfort it is to the patient, as well as a convenience, to close an ulcer of long standing without depriving him of one day's loss of time.

*Acute Arthritis:*—Whether the inflammation be the result of a trauma or from rheumatism, the bandage rapidly restores the joint to its normal condition. I have had the most gratifying results in dislocations of the elbow associated with great injury to the soft parts. With our best efforts, heretofore, anchylosis has too often resulted. The elastic controls the blood supply, and hence the degree of inflammation. It is kept at the reparative point, this side of that of suppuration or of too much plasticity. Passive motion for the protection of the function of the joint can be much sooner instituted.

*Weight, Length and Strength of the Bandage.*—Some care is necessary in selecting the bandage, as to its length, width and weight, and mode of application.

1. The limb should not be doubly covered, except where the bandage laps.

2. The ordinary bandage of Martin is not too strong for the inferior extremities, but one of lighter make is better adapted to the arm and forearm.

3. The "rubber dam" is sufficiently heavy for the fingers; a thicker one acts too powerfully on the circulation, and makes too great pressure on the granulations.

4. Children require a lighter bandage than adults. The variety suited to the arm of the adult will be heavy enough for the leg of the child.

5. The width of the bandage must be regulated according to the part to be covered; it may range from one to three and a half inches.

*Mode of Application.*—But little force must be used in applying the bandage. As I said above, you desire to control, to regulate the circulation, not to prevent or obstruct it. In adjusting the heaviest elastic upon the leg, the weight is almost sufficient. Irregular pressure must be studiously avoided, the turns must press at all points equally. The sensations of the patient will often be a reliable guide in adjustment.

*Inflammation of Ankle with a Strumous Tendency.*—This little boy has an affection of the ankle, the result of an injury; the joint was struck several weeks ago, it was puffy and painful when I first saw it, and there was slight effusion in the sac.

The bandage, which you now see me apply, weighs one ounce and a half; it is too inches wide and three and a half yards long. I have been using it for less than one week, and you see the joint is almost if not quite normal. This change has been the result of the bandage alone; without it the low form of inflammation which had been established would have gone on for months, and perhaps for years, and have finally resulted in the destruction of the functions of the joint. Although the result of a trauma, the case impressed me as tending to a strumous degeneration.

This boy has not been restrained in his movements since he has been wearing the bandage. When presented at my office, one week ago, he was lame, and the joint tender; you now see that his gait is without a limp, and I handle the limb without giving him pain. Over the bandage he wears his stocking and shoe.

I have now, gentlemen, given you somewhat of my experience in the use of this recently introduced appliance. I think it would be difficult to overrate its usefulness. As I said in the beginning of the lecture, it is destined to modify the treatment of chronic and acute disorders of the extremities and it may have a wider range of utility.—*Good Samar. Hosp. Rep., Lancet and Clinic.*

